# How school climate influences teacher innovation: the chain mediation role of teacher autonomy and self-efficacy

**DOI:** 10.3389/fpsyg.2026.1834315

**Published:** 2026-06-22

**Authors:** Chiyu Zhou, Yumeng Liu, Jing Dong, Shuping Liu

**Affiliations:** 1 Faculty of Education, Beijing Normal University, Beijing, China; 2 Research Base for Primary and Early Childhood Education of Hainan Province, Qiongtai Normal University, Haikou, China

**Keywords:** psychological empowerment theory, school climate, self-efficacy, teacher autonomy, teacher innovation

## Abstract

**Introduction:**

Teacher innovation is a key manifestation of teachers’ professional competence and is essential for school development and student learning. Although previous research has identified a positive association between school climate and teacher innovation, the underlying mechanisms remain insufficiently explored, particularly in the Chinese context.

**Methods:**

Drawing on psychological empowerment theory, this study examines the relationship between school climate and teacher innovation and tests a sequential mediation model involving teacher autonomy and self-efficacy. Data were collected from 3,976 secondary school teachers in Shanghai, China, using the TALIS 2018 database. Structural equation modeling was employed for data analysis.

**Results:**

The results indicate that school climate has a significant positive effect on teacher innovation. In addition, teacher autonomy and self-efficacy sequentially mediate this relationship, forming a chain pathway: School Climate → Teacher Autonomy → Self-Efficacy → Teacher Innovation. Specifically, a positive school climate enhances teacher autonomy, which subsequently strengthens teachers’ self-efficacy and ultimately promotes innovative behavior.

**Discussion:**

These findings clarify the mechanisms linking school climate to teacher innovation and extend psychological empowerment theory by highlighting the sequential interplay between autonomy and competence in shaping teachers’ innovative behavior.

## 1 Introduction

Teachers play a central role in shaping classroom practices and educational quality (Thurlings et al., 2015). As a key dimension of teachers’ professional competence, teacher innovation has attracted increasing attention from both policymakers and school administrators in recent years. Teacher innovation not only enhances instructional quality but also fosters students’ creativity, problem-solving abilities, and critical thinking, thereby contributing to long-term educational development (Zhu et al., 2013; Serdyukov, 2017). In this sense, teacher innovation can be understood as teachers’ proactive development and creative application of new pedagogical ideas, methods, and practices in response to evolving educational demands (Anderson et al., 2014). It encompasses both teachers’ willingness to innovate and their actual implementation of innovative teaching practices, reflecting a multidimensional construct involving instructional design, teaching methods, and assessment practices (Ainley and Carstens, 2018; Li and Zhu, 2022). Fundamentally, teacher innovation makes teaching more engaging, enhances the motivation of all participants in the learning process, promotes student development, and enables students to better cope with learning challenges and future demands (Karolčík and Marková, 2025; Cao et al., 2025; Garzón Artacho et al., 2020).

A growing body of research has examined the factors influencing teacher innovation from both individual and organizational perspectives. At the individual level, factors such as teacher collaboration (Pan et al., 2024), self-efficacy (Hsiao et al., 2011), job satisfaction (Blömeke et al., 2021), and professional autonomy (Lin, 2022) have been shown to positively predict innovative behavior. At the organizational level, distributed leadership (Buyukgoze et al., 2024; Wang and Bai, 2025) and school climate (Balkar, 2015; Zhang et al., 2024; Kundu and Roy, 2023; Cai and Tang, 2021; Fidan and Oztürk, 2015) are widely recognized as critical contextual conditions shaping teacher innovation. School climate—encompassing leadership practices, collegial relationships, work environment, and organizational support—has been consistently identified as a key factor that not only directly promotes innovative teaching practices but also indirectly influences teachers’ beliefs, motivations, and attitudes toward innovation (Balkar, 2015; Beets et al., 2008).

Despite these advances, existing studies exhibit two important limitations. First, while prior research has established a positive relationship between school climate and teacher innovation, it has predominantly focused on direct effects, with insufficient attention to the underlying psychological mechanisms through which school climate translates into innovative behavior. As a result, the “black box” linking organizational context to teacher innovation remains inadequately explained. Second, although some studies have examined individual factors such as autonomy and self-efficacy separately, there is a lack of integrative frameworks that explain how these psychological resources operate jointly within a coherent theoretical model. This limits a deeper understanding of how environmental conditions are internalized into teachers’ psychological states and subsequently transformed into innovative practices.

Psychological empowerment theory provides a valuable lens to address these gaps. This theory conceptualizes empowerment as a set of intrinsic motivational states, typically including dimensions such as autonomy (self-determination) and competence (self-efficacy). From this perspective, school climate can be seen as an important contextual resource that shapes teachers’ psychological empowerment by enhancing their sense of autonomy and efficacy. Specifically, a supportive and trust-based school climate can enhance teachers’ professional autonomy by granting greater discretion in instructional design, classroom management, and curriculum decision-making (Ozdemir and Cakalci, 2022; Thurlings et al., 2015). At the same time, collaborative and resource-rich school environments foster communication and cooperation among teachers, enabling the sharing of experiences and resources, which strengthens teachers’ confidence in their teaching capabilities and enhances self-efficacy (Zysberg and Schwabsky, 2021). These psychological states, in turn, serve as important drivers of innovative behavior (Buske, 2018; Tschannen-Moran and Hoy, 2001). Moreover, psychological empowerment theory implies a potential sequential process in which enhanced autonomy may further reinforce teachers’ sense of competence, forming a chain mediation mechanism linking school climate to teacher innovation.

Furthermore, the Chinese educational context provides a particularly meaningful setting for examining these mechanisms. In recent years, China has undergone significant educational reforms aimed at shifting from an “exam-oriented” approach to a “competency-based” approach, placing increasing emphasis on teaching quality and innovation. Policies such as the “Double Reduction” initiative have further intensified expectations for instructional improvement and teacher innovation. At the same time, Chinese schools are characterized by hierarchical and collectivist organizational cultures, which may simultaneously constrain individual autonomy and promote collaboration and support. Previous studies suggest that while teachers’ professional autonomy in Chinese schools still has room for improvement, school support, colleague collaboration, and principals’ instructional leadership exert significant influences on teachers’ psychological empowerment, self-efficacy, and innovative behavior (Liu and Hallinger, 2018; Cai and Tang, 2021). However, empirical evidence on the underlying mechanisms remains limited, particularly in large-scale quantitative studies.

To address these gaps, this study draws on psychological empowerment theory to examine the relationship between school climate and teacher innovation in China, with a particular focus on the mediating roles of teacher autonomy and self-efficacy. By integrating organizational and psychological perspectives, this study aims to uncover the internal mechanisms through which school climate influences teacher innovation and to extend the application of psychological empowerment theory in educational research.

Accordingly, this study addresses the following research questions:

*RQ1*: Does school climate influence teacher innovation among Chinese teachers?

*RQ2*: Do teacher autonomy and teacher self-efficacy mediate the relationship between school climate and teacher innovation?

### 1.1 Psychological empowerment theory

Psychological empowerment theory was first proposed by Thomas and Velthouse (1990) and later developed into a mature motivational framework by Spreitzer (1995). The theory emphasizes that empowerment is not merely an external managerial practice but a positive psychological state experienced by individuals within organizational contexts, reflecting employees’ perceptions, attitudes, and motivational experiences toward their work roles. Unlike traditional “structural empowerment,” psychological empowerment focuses on individuals’ intrinsic perceptions and subjective experiences, and is thus considered an intrinsically motivated psychological mechanism. This mechanism is further divided into four dimensions: meaning, competence, self-determination, and impact. According to psychological empowerment theory, psychological empowerment does not occur in isolation; rather, it is an internally motivated state triggered by external organizational contexts. Such a positive internal state promotes initiative, creativity, and innovative behaviors (Thomas and Velthouse, 1990; Spreitzer, 1995). From this theoretical perspective, school climate, as an external organizational context, can facilitate teachers’ instructional innovation by stimulating self-determination (e.g., teacher autonomy) and competence (e.g., self-efficacy). Therefore, psychological empowerment theory provides a useful framework for this study to examine the relationships among school climate, teacher autonomy, self-efficacy, and teacher instructional innovation, enhancing our understanding of how school climate fosters teacher innovation.

### 1.2 School climate and teachers’ innovation

School climate is a multifaceted concept encompassing academic climate, community relations, campus safety, and institutional environment. The academic climate refers to a learning-oriented context, including teaching, learning, and professional development; the community dimension reflects interpersonal relationships among teachers, students, families, and the broader school community; campus safety refers to equitable and effective disciplinary policies and the protection of teachers’ and students’ physical and psychological well-being; and the institutional environment concerns organizational structures, facilities, and resource availability (Wang and Degol, 2016).

A positive school climate is an effective means of achieving organizational goals (Altuntaş et al., 2020), as it can motivate members and foster emotional attachment and pride in the school among principals, teachers, and other staff (Işık, 2020; Atik and Güneri, 2016). Furthermore, a supportive climate enhances schools’ openness to reform and innovation (Bodur and Argon, 2019).

Since teachers’ innovative instructional behaviors are influenced by external conditions, schools—as the primary workplace and professional development setting—play a decisive role. Bronfenbrenner and Evans (2000a,b) proposed the ecological systems theory, asserting that individuals’ behaviors and development are shaped by their surrounding environments. As a key component of the mesosystem, school climate significantly impacts teacher innovation. Learning- and development-oriented organizational climates stimulate teachers to explore new instructional methods and strategies (Thurlings et al., 2015). Such environments help establish organizational identification, enhance teachers’ sense of responsibility, and motivate greater engagement in innovative teaching (Fujita et al., 2018). Additionally, supportive climates facilitate teachers’ adoption of new instructional technologies and methods (Chou et al., 2019). Recent studies further indicate that school climate not only directly predicts instructional innovation but can also indirectly influence innovation via teaching efficacy (Zhang et al., 2024).

Based on the above analysis, the following hypothesis is proposed:

*H1*: School climate is positively correlated with and significantly predicts teacher innovation.

### 1.3 The mediating role of teacher autonomy

Teacher autonomy refers to the rights and freedom that teachers possess within their professional domains (Pearson and Moomaw, 2005), with its core lying in the reasonable exercise of decision-making authority and responsibility (Yorulmaz and Çolak, 2023). Previous research indicates that teacher autonomy serves multiple functions, including the development of instructional and learning activities, the adoption of diversified teaching methods, and the promotion of professional growth (Xia et al., 2023; Jang et al., 2024; Demir and Kalman, 2025). Empirical studies further demonstrate that autonomy affects a range of innovation and school-level variables, such as job satisfaction (Xie and Zou, 2025), professionalism (Pearson and Moomaw, 2005), motivation (Fradkin-Hayslip, 2021), self-efficacy (Choi and Mao, 2021), and psychological empowerment (Yorulmaz et al., 2018).

Teacher autonomy is regarded as a key component of teacher quality (Çolak, 2024). Teachers with high autonomy are generally more motivated and willing to engage in educational innovation (Zhang et al., 2024). A study of 473 Chinese university teachers found that autonomy is significantly positively correlated with innovative performance and teaching satisfaction (Han et al., 2021). Results from the 2022 Program for International Student Assessment (PISA) also indicate that teachers’ emphasis on innovative teaching practices is closely dependent on the level of autonomy granted to them across different systems (OECD, 2024).

School climate plays a critical role in promoting teacher autonomy. A democratic and sincere school climate can significantly enhance teachers’ sense of self-determination (Akgöz et al., 2024). For example, a study based on data from 1,276 teachers across 44 kindergartens in China found that fostering a more supportive organizational climate at the school level can significantly improve teacher autonomy (Wang et al., 2026). In addition, a study using TALIS 2018 data from South Africa showed that school climate can significantly enhance teacher autonomy and, in turn, predict teacher job satisfaction (Oduro et al., 2025).

Based on the above analysis, this study proposes the following hypotheses:

*H2a*: School climate is positively correlated with teacher autonomy.

*H2b*: Teacher autonomy is positively correlated with teacher innovation.

*H2c*: Teacher autonomy mediates the relationship between school climate and teacher innovation.

### 1.4 The mediating role of teachers’ self-efficacy

Bandura (1997) defined self-efficacy as individuals’ beliefs in their capabilities to organize and execute actions required to achieve desired outcomes. It reflects self-assessment of task-specific competence independent of others’ performance (Tschannen-Moran and Hoy, 2007). Self-efficacy is primarily developed through mastery experiences, physiological and emotional states, and vicarious learning from others’ successes or failures (Bandura, 1997). Gibson and Dembo (1984) further distinguished teacher efficacy into two dimensions: Personal Teaching Efficacy (PTE) and General Teaching Efficacy (GTE), the former representing teachers’ confidence in their own abilities and the latter reflecting beliefs about their overall influence on student outcomes.

In educational contexts, instructional innovation is inherently complex and uncertain, especially within performance-oriented systems, making self-efficacy a key psychological factor influencing teachers’ choices. Teachers with higher self-efficacy are more willing to adopt new instructional approaches (Çimen et al., 2023), integrate innovative ideas and tools into practice (Zhang et al., 2024), and demonstrate greater resilience when facing setbacks (Cai and Tang, 2021). Empirical studies also confirm that innovative self-efficacy is positively associated with actual innovation behaviors (Gkontelos et al., 2023; Teng et al., 2024), and is positively associated with innovative teaching while also moderating the relationship between teacher–student relationships and such practices, as shown in TALIS 2018 Shanghai data (Wang, 2025).

School climate significantly affects teacher self-efficacy (Shah et al., 2022; de Arellano et al., 2023). It plays a crucial role in strengthening teachers’ confidence in their own abilities, which is essential for their professional development and effectiveness (Alshuhumi et al., 2025). Teachers’ innovative instructional practices and the creation of sustainable learning environments can enhance students’ academic motivation and psychological well-being, both of which are influenced by teachers’ self-efficacy (Nasser et al., 2024). Empirical evidence indicates that teacher self-efficacy serves as a mediator between school climate and instructional innovation (Alshuhumi et al., 2025). Additionally, a study on newly hired teachers at Southeast Asian Institute of Applied Technology (SEAIT) during the first semester of the 2025–2026 academic year found that school cultural climate has a significant effect on teaching efficacy (Jorolan et al., 2026).

Based on the above analysis, this study proposes the following hypotheses:

*H3a*: School climate is positively correlated with teachers’ self-efficacy.

*H3b*: Teachers’ self-efficacy is positively correlated with teacher innovation.

*H3c*: Teachers’ self-efficacy mediates the relationship between school climate and teacher innovation.

### 1.5 The chain-mediating role of teacher autonomy and self-efficacy

Teacher autonomy is a foundational factor affecting instructional practices and may positively influence teacher self-efficacy. When teachers have sufficient autonomy, they can set instructional goals, design lesson plans, and choose teaching methods that best meet students’ developmental needs, thereby enhancing work meaningfulness and overall satisfaction (Xia et al., 2023), which in turn strengthens teaching efficacy. Research has shown that autonomy promotes reflective dialog through the enhancement of self-efficacy (Valckx et al., 2020), and self-efficacy mediates the relationship between autonomy and psychological well-being (Peng et al., 2022). Similarly, studies indicate that teacher autonomy positively predicts self-efficacy (Özdemir et al., 2024). Furthermore, research has demonstrated that teacher autonomy and self-efficacy jointly play a significant chain-mediating role between principals’ distributed leadership and teacher innovation (Wang and Bai, 2025). This provides a useful basis for formulating reasonable hypotheses in the present study, indicating a close relationship between teacher autonomy and self-efficacy.

Based on the above analysis, this study proposes the following hypotheses:

*H4a*: Teacher autonomy is significantly and positively associated with teachers’ self-efficacy.

*H4b*: Teacher autonomy and teachers’ self-efficacy jointly play a chain-mediating role between school climate and teacher innovation.

**Figure 1:**
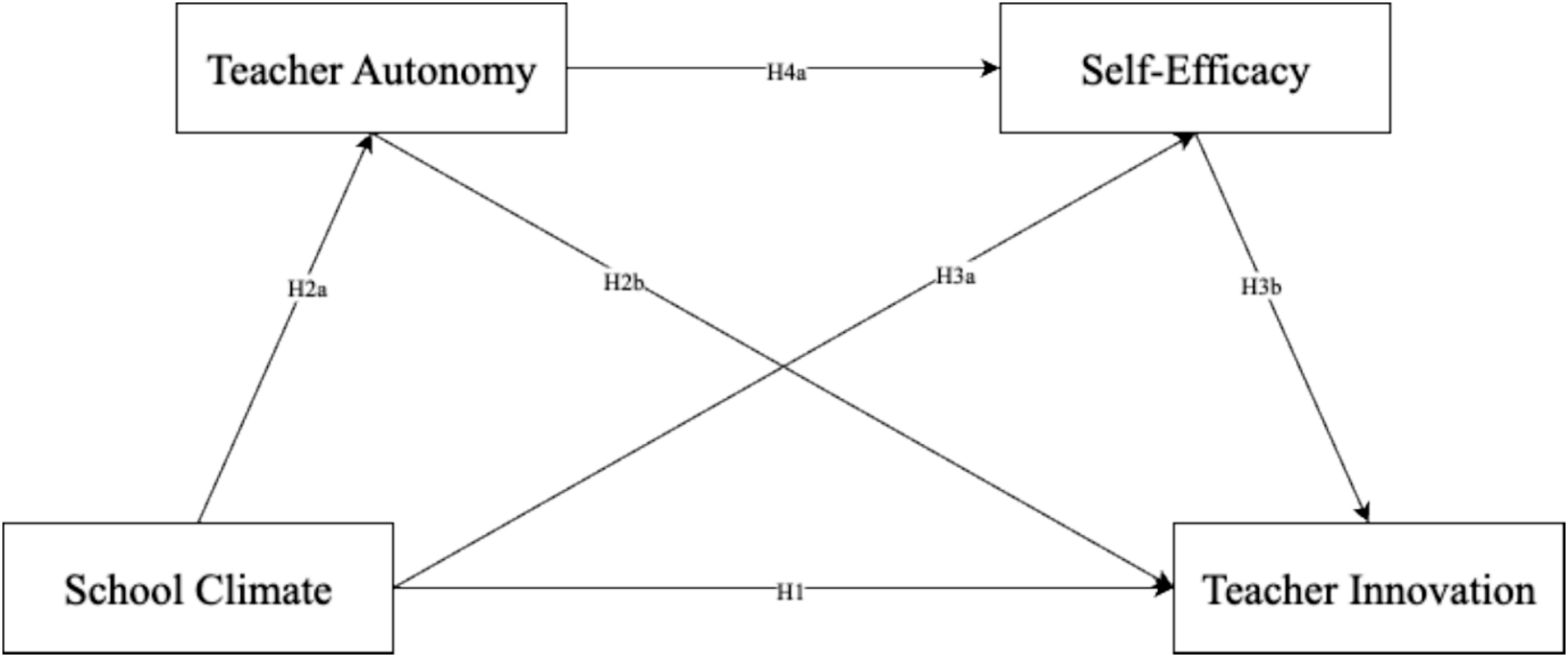
Research framework.

Based on the research hypotheses presented above, a research framework is proposed, as illustrated in Figure 1.

## 2 Materials and methods

### 2.1 Data resource

Data for this study were drawn from the 2018 Teaching and Learning International Survey (TALIS) database. Conducted by the Organization for Economic Cooperation and Development (OECD), TALIS is one of the largest international surveys in the field of education, aiming to understand the working conditions of teachers and principals as well as the learning environments of schools. The 2018 TALIS represented the third round of the survey was conducted across 48 countries and regions, involving approximately 260,000 teachers and 15,000 schools. And the items and measured in the questionnaires vary across different rounds. The TALIS 2018 teacher questionnaire measures teachers’ professional practices across multiple dimensions, including school climate, teacher autonomy, teacher innovation.

In total, 3,976 secondary school teachers from Shanghai, China, participated in this TALIS survey. The demographic characteristics of the Shanghai sample are as follows. In terms of gender, 2,941 participants were female (74.0%) and 1,035 were male (26.0%). As for teacher age, 119 teachers were under 25 years old (3.0%), 534 were aged 25–29 (13.4%), 1,316 were aged 30–39 (33.1%), 1,418 were aged 40–49 (35.7%), 566 were aged 50–59 (14.2%), and 22 were 60 years old or above (0.6%).

### 2.2 Measure

The measurement scales used in this study were derived from the items of the TALIS 2018 teacher questionnaire.

#### 2.2.1 School climate

The school climate scale consists of five items, with representative examples including: “Teachers and students usually get on well with each other” and “Teachers can rely on each other.” All items were rated on a four-point Likert scale ranging from 1 (strongly disagree) to 4 (strongly agree), with higher scores indicating a more positive perception of the school climate by teachers. The scale demonstrated good reliability, with a Cronbach’s alpha coefficient of 0.929, with fit indices of χ^2^/df = 7.216, CFI = 0.996, TLI = 0.985, RMSEA = 0.043, and SRMR = 0.008.

#### 2.2.2 Teacher autonomy

The teacher autonomy scale consists of five items, with representative examples including: “Determining course content,” “Selecting teaching methods,” and “Assessing students’ learning.” Participants rated these items on a four-point Likert scale ranging from 1 (strongly disagree) to 4 (strongly agree), with higher scores indicating a higher level of teacher autonomy. The scale demonstrated good reliability, with a Cronbach’s alpha coefficient of 0.923, with fit indices of χ^2^/df = 6.888, CFI = 0.990, TLI = 0.975, RMSEA = 0.046, and SRMR = 0.013.

#### 2.2.3 Self-efficacy

The self-efficacy scale consists of three dimensions: classroom management, instructional self-efficacy, and student engagement self-efficacy. Each dimension consists of four items, for example, “Control disruptive behavior in the classroom,” “Use a variety of assessment strategies,” and “Get students to believe they can do well in school work.” Participants rated the items on a four-point Likert scale ranging from 1 (strongly disagree) to 4 (strongly agree). Higher scores indicate greater teacher self-efficacy. The Cronbach’s alpha coefficients for the three dimensions were 0.919, 0.890, and 0.892, respectively, with an overall scale reliability of 0.956. CFA yielded acceptable fit indices (χ^2^/df = 8.250, CFI = 0.983, TLI = 0.974, RMSEA = 0.047, SRMR = 0.020), supporting the scale’s reliability and validity. In the structural equation model, teacher self-efficacy was specified as a second-order reflective construct indicated by the three dimensions.

#### 2.2.4 Teacher innovation

The teacher innovation scale consists of four items, with representative examples including: “Most teachers in this school strive to develop new ideas for teaching and learning” and “Most teachers in this school are open to change.” Participants rated the items on a four-point Likert scale ranging from 1 (strongly disagree) to 4 (strongly agree). Higher scores reflect a higher level of teacher innovation. The scale showed excellent reliability, with a Cronbach’s alpha coefficient of 0.949, with fit indices of χ^2^/df = 6.292, CFI = 0.997, TLI = 0.990, RMSEA = 0.040, and SRMR = 0.006.

### 2.3 Data analysis

This study employed SPSS 27.0 and Mplus 8.3 for statistical analysis and data processing. Missing data were handled using full information maximum likelihood (FIML) estimation, which utilizes all available information without listwise deletion. First, descriptive statistics and one-way analysis of variance (ANOVA) were performed in SPSS 27.0 on the teacher innovation data from Shanghai to examine potential differences across various groups. Second, to ensure the reliability and validity of the study, assessments of common method bias and multicollinearity were conducted, followed by Pearson correlation analyses among the key variables. All Likert-type items were treated as continuous variables, and maximum likelihood estimation was used in Mplus 8.3. Finally, based on the proposed research hypotheses, a structural equation model (SEM) was constructed, and both model fit and mediating effects were examined using Mplus 8.3 to test the validity of the hypothesized relationships.

Given the nested structure of TALIS data (teachers within schools), intraclass correlation coefficients (ICCs) were computed for all focal variables. The ICCs were 0.063 for school climate, 0.021 for teacher autonomy, 0.045 for self-efficacy, and 0.055 for teacher innovation, indicating that between-school variance accounted for only 2.1–6.3% of the total variance. Given these relatively low values, single-level SEM was deemed appropriate. Univariate skewness and kurtosis values for all indicators fell within acceptable thresholds, supporting the assumption of approximate normality. No significant outliers were identified based on standardized scores and descriptive inspection of the data. Accordingly, maximum likelihood estimation with 5,000 bootstrap resamples was employed to obtain robust standard errors and confidence intervals for model fit and mediating effects.

## 3 Results

### 3.1 Common method bias test

To assess potential common method bias, three complementary approaches were employed. First, Harman’s single-factor test was conducted. The results showed that the variance explained by the first factor without rotation was 41.128%, which is below the 50% threshold (Podsakoff and Organ, 1986). Second, a common latent factor (CLF) test indicated that the CLF accounted for only 4.22% of the variance in the indicators, and all item loadings on their theoretical constructs remained stable with and without the CLF, with the maximum change being 0.06 (Podsakoff et al., 2003). Third, collinearity diagnostics with teacher innovation as the dependent variable yielded Variance Inflation Factor (VIF) values of 1.289, 1.287, and 1.269 for school climate, teacher autonomy, and self-efficacy, respectively, all below 5. Taken together, these results indicate that common method bias is not a serious concern in this study.

### 3.2 Descriptive statistics and correlations

The means, standard deviations, and Pearson correlation coefficients among school climate, teacher autonomy, self-efficacy, teacher innovation, gender and teacher age are presented in Table 1. All pairs of the focal variables exhibited significant positive correlations, providing a solid foundation for subsequent analyses. All constructs demonstrated good convergent validity, with composite reliability (CR) values of 0.924 (school climate), 0.921 (teacher autonomy), 0.952 (self-efficacy), and 0.949 (teacher innovation), and average variance extracted (AVE) values of 0.710, 0.702, 0.621, and 0.824, respectively. Discriminant validity was further supported, as the square root of each construct’s AVE (shown on the diagonal of Table 1) exceeded its correlations with other constructs.

**Table 1:** Descriptive statistics and correlation analysis (*N* = 3,976).

Variable	*M*	SD	1	2	3	4	5	6
1. GN	1.260	0.439	1					
2. TY	3.464	1.008	0.091***	1				
3. SC	16.738	2.429	−0.010	−0.080***	0.843			
4. TA	16.955	2.540	−0.059***	−0.106***	0.399***	0.838		
5. SE	39.745	6.495	−0.012	0.124***	0.381***	0.386***	0.788	
6. TI	12.695	2.389	−0.025	−0.052***	0.482***	0.280***	0.263***	0.908

M, mean; SD, Standard Deviation; GN, Gender; TY, Teacher Age; SC, School Climate; TA, Teacher Autonomy; SE, Self-Efficacy; TI, Teacher Innovation.

*p < 0.05, **p < 0.01, ***p < 0.001.

### 3.3 Mediation effect test

A structural equation model was constructed with school climate as the independent variable, teacher innovation as the dependent variable, and teacher autonomy and self-efficacy as sequential mediators. The hypothesized model was tested as specified *a priori*; no post-hoc modifications were made based on modification indices. The model fit indices indicated a good fit: χ^2^/df = 5.499, CFI = 0.973, TLI = 0.968, RMSEA = 0.037, and SRMR = 0.027. Although the χ^2^/df value is above the more conservative threshold, this is likely due to the well-documented sensitivity of the chi-square statistic to large sample sizes, which tends to inflate the χ^2^ value (Sathyanarayana and Mohanasundaram, 2024). Therefore, greater emphasis is placed on alternative fit indices, all of which indicate a good model fit.

**Figure 2:**
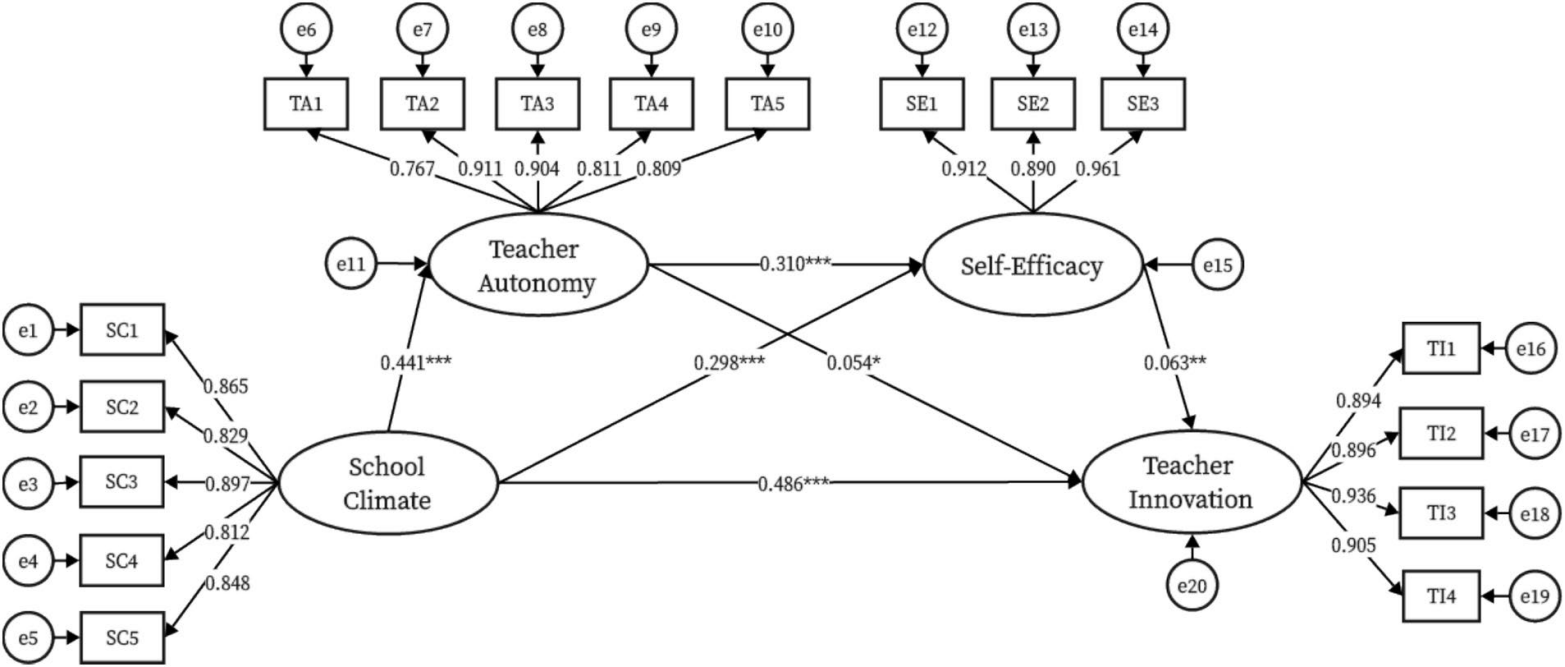
Mediation effect model.

As shown in Figure 2, school climate had a significant positive effect on teacher innovation (β = 0.486, *p* < 0.001), teacher autonomy (β = 0.441, *p* < 0.001), and self-efficacy (β = 0.298, *p* < 0.001). Teacher autonomy positively influenced teacher innovation (β = 0.054, *p* < 0.05) and self-efficacy (β = 0.310, *p* < 0.001). Self-efficacy also had a significant positive effect on teacher innovation (β = 0.063, *p* < 0.01).

Using Mplus 8.3, the mediation effects of teacher autonomy and self-efficacy were tested via the bootstrap method with 5,000 bootstrap samples. A mediation effect was considered significant if the 95% confidence interval did not include zero. The results of the mediation test are shown in Table 2. The total effect of school climate on teacher innovation was significant (β = 0.537, 95% CI [0.499, 0.572], *p* < 0.001). The indirect effect via teacher autonomy (β = 0.024, 95% CI [0.004, 0.044], *p* < 0.05) and the indirect effect via self-efficacy (β = 0.019, 95% CI [0.006, 0.033], *p* < 0.01) were both significant, as was the chain indirect effect through teacher autonomy and self-efficacy (β = 0.009, 95% CI [0.003, 0.015], *p* < 0.01). The direct effect of school climate on teacher innovation remained significant (β = 0.486, 95% CI [0.439, 0.533], *p* < 0.001). These results indicate that teacher autonomy and self-efficacy partially mediate the relationship between school climate and teacher innovation. Research hypotheses 1, 2a, 2b, 2c, 3a, 3b, 3c, 4a, and 4b were thus supported.

**Table 2:** Mediation effects and effect sizes (*N* = 3,976).

Effect Type	Path	Effect	Bootstrap 95% CI		*p*	Ratio
			Lower	Upper		
Direct effect	SC-TI	0.486	0.439	0.533	0.000	90.5%
Indirect effect 1	SC-TA-TI	0.024	0.004	0.044	0.024	4.47%
Indirect effect 2	SC-SE-TI	0.019	0.006	0.033	0.006	3.54%
Indirect effect 3	SC-TA-SE-TI	0.009	0.003	0.015	0.006	1.68%
Indirect effect	–	0.051	0.026	0.075	0.000	9.50%
Total effect	–	0.537	0.499	0.572	0.000	100%

SC, School Climate; TA, Teacher Autonomy; SE, Self-Efficacy; TI, Teacher Innovation.

## 4 Discussion

This study examined how school climate influenced teacher innovation through the mediating roles of teacher autonomy and self-efficacy, using data from 3,976 secondary school teachers in Shanghai collected by the 2018 Teaching and Learning International Survey (TALIS). The findings provide empirical evidence on how environmental and psychological factors jointly facilitate instructional innovation.

First, the results indicated that school climate was a significant positive predictor of teacher innovation. Teachers who perceived their schools as collaborative, fair, and supportive were more likely to engage in innovative instructional practices. This finding aligned with prior research emphasizing the critical role of organizational climate in shaping teachers’ attitudes, motivation, and behavior (Ozdemir and Cakalci, 2022; Balkar, 2015; Kundu and Roy, 2023). Empirical evidence suggested that innovation thrived in open and trusting environments, where teachers who perceived support from colleagues and administrators were more willing to take instructional risks and challenge conventional practices (Thurlings et al., 2015). Conversely, highly bureaucratic or low trust schools could constrain teacher initiative, limiting innovation (Zhao and Jin, 2023). Notably, Chinese basic education has historically been characterized by a highly centralized management system, with standardized curricula, examinations, and school evaluations (Hawkins, 2000). While such a system might be perceived as limiting teacher autonomy, the findings showed that mesoscale interventions—specifically school climate improvements—could activate teacher innovation even within a highly regulated context. This demonstrates that organizational environments can buffer structural constraints, providing psychological safety and space for creative action.

Second, the study showed that teacher autonomy partially mediated the relationship between school climate and innovation. In other words, a supportive school climate enhanced teacher autonomy, which in turn stimulated engagement in innovative instructional practices. Scholarly work in recent years has further emphasized that teacher autonomy is a core component of teacher quality and a key predictor of innovative practices (Çolak, 2024; OECD, 2024). School climate was positively associated with teacher autonomy, corroborating prior studies (Shah et al., 2022; de Arellano et al., 2023). Evidence across diverse educational contexts has further confirmed this relationship (Akgöz et al., 2024; Wang et al., 2026; Oduro et al., 2025). In schools emphasizing openness and independence, teachers were more involved in decision-making, instructional planning, and curriculum design, thereby enhancing flexibility and professional agency. Furthermore, teacher autonomy positively predicted instructional innovation, consistent with previous research (Li and Zhu, 2022; Prasetyo and Purwoto, 2024). Empirical findings in related studies also suggest that teachers with higher autonomy are more motivated and more willing to engage in innovative teaching practices (Zhang et al., 2024; Teng et al., 2024). Autonomy served as an internal motivator, encouraging teachers to explore new instructional ideas, methods, and tools, improving teaching quality and enabling teachers to respond effectively to complex educational challenges. In the Chinese context, teacher autonomy had traditionally been constrained by centralized curricula and exam-driven accountability structures. However, reforms such as diversified curricula, formative assessment, and school-based management had increased decision making latitude, and the findings provided empirical evidence that such autonomy positively and measurably influenced innovation, highlighting the importance of continued empowerment policies.

Third, the results indicated that self-efficacy also significantly mediated the relationship between school climate and teacher innovation. In other words, school climate not only directly facilitated innovative behavior but also indirectly promoted innovation by enhancing teachers’ self-efficacy. A growing stream of research has extended this perspective by demonstrating that self-efficacy not only predicts innovation adoption but also shapes persistence in the face of instructional challenges (Teng et al., 2024; Gkontelos et al., 2023). Teachers with higher self-efficacy were more likely to persist in innovation when facing challenges and to experiment with new instructional approaches (Çimen et al., 2023). The results confirmed these theoretical predictions, showing that teachers with greater self-efficacy were more proactive in implementing novel methods and exhibited stronger adaptive capabilities in classroom management and student engagement. Thus, self-efficacy served as a key psychological bridge linking supportive school features with innovative teaching behaviors. Specifically, school climate was positively associated with teacher self-efficacy, consistent with Hosford and O'Sullivan (2016) and Lu et al. (2015). Emerging evidence further supports that supportive school environments enhance teachers’ confidence and perceived competence, thereby facilitating innovative practices (Alshuhumi et al., 2025; Jorolan et al., 2026). In supportive environments, teachers received recognition and positive feedback, fostering stronger self-efficacy, which in turn enhanced innovation (Cai and Tang, 2021; Zhang et al., 2024). Teacher innovation depended not only on external institutional conditions but also on teachers’ beliefs in their abilities and value. When teachers believed they could meaningfully impact student learning, they were more likely to engage proactively in innovation and to view it as integral to professional growth. Although teacher autonomy and self-efficacy jointly mediate the relationship between school climate and teacher innovation, the direct effect of school climate on teacher innovation remains statistically significant. This suggests that the proposed sequential mediation model does not fully capture all underlying mechanisms. One possible explanation is that school climate may also influence teacher innovation through additional psychological or contextual pathways not included in the present model. For instance, factors such as psychological capital (Zhou and Ismail, 2025), teaching efficacy (Zhang et al., 2024) or organizational trust may directly shape teachers’ willingness to engage in innovative practices beyond individual autonomy and self-efficacy. In addition, institutional resources and policy support at the school level may also play a direct role in fostering innovation. These unobserved mechanisms may collectively account for the remaining direct effect. Future research is encouraged to incorporate these additional mediating or moderating variables to further refine the explanatory power of the model and provide a more comprehensive understanding of how school climate influences teacher innovation.

Finally, this study identified a chain-mediating pathway of “school climate → teacher autonomy → self-efficacy → teacher innovation,” thereby enriching the existing theoretical framework. The findings suggested that school climate enhanced teacher autonomy, which in turn strengthened self-efficacy and ultimately promoted innovation. This insight advanced the understanding of psychological empowerment theory, indicating that self-determination and competence may not operate in parallel but in a sequentially reinforcing manner. Teacher autonomy and self-efficacy were interlinked: autonomy, as an external structural condition, provided decision-making latitude, while self-efficacy, as an internal psychological resource, enabled and motivated teachers to exercise this autonomy. Together, they formed a dual mechanism driving innovation. This process could be conceptualized as developmental: supportive school climates promoted autonomy; enhanced autonomy increased teachers’ sense of competence and confidence; heightened self-efficacy translated into innovative instructional practices.

It is important to note that several paths in the model are statistically significant but relatively small in magnitude. Although the standardized coefficients of these paths are modest, their value lies not only in statistical significance but also in their conceptual and practical implications. From a practical perspective, while these effects may be limited at the individual level, they can accumulate over time and produce meaningful impacts at the organizational level within large-scale educational systems. The findings suggest that the influence of school climate does not operate through a single direct step, but rather unfolds through a layered process of internalization. A positive school climate first enhances teachers’ sense of autonomy, which in turn strengthens their self-efficacy, ultimately promoting innovative instructional practices. This progressive process provides empirical support for psychological empowerment theory, indicating that structural environmental factors can be gradually internalized as individual psychological resources and subsequently translated into behavioral outcomes. Therefore, although the standardized coefficients of these paths are relatively small, their contribution lies in theoretically revealing the dynamic interconnections among different dimensions of psychological empowerment. In this sense, the value of these findings is reflected in their explanatory refinement, offering a more nuanced understanding of how organizational environments are translated into teacher innovation through step-by-step psychological mechanisms. At the same time, the results suggest that interventions targeting a single psychological factor may have limited effects, whereas fostering the coordinated development of both teacher autonomy and self-efficacy may be more effective in promoting teacher innovation. Overall, this study clarified the specific pathways through which school climate influenced teacher innovation, highlighting the critical interplay between organizational environment and psychological empowerment even within a centralized and standardized education system.

## 5 Theoretical and practical implications

This study makes several theoretical contributions. First, it deepens the understanding of the relationship between the autonomy and competence dimensions in psychological empowerment theory. Traditional psychological empowerment theory did not explicitly clarify the relationship between these two dimensions (Thomas and Velthouse, 1990; Spreitzer, 1995). The present study found that in a supportive school climate, teacher autonomy was first activated, which subsequently enhanced self-efficacy, thereby promoting innovative behavior. This sequential mechanism suggests that autonomy and competence may not operate in parallel but rather in a progressive manner. Second, the study provides empirical evidence within the Chinese educational context. Despite the system’s emphasis on uniformity and standardization, the findings indicate that a positive school climate can enhance teacher autonomy and self-efficacy, thereby fostering innovation. This underscores the critical role of the mesoscale school environment in facilitating teacher innovation even under macro-level institutional constraints, offering support for the applicability of psychological empowerment theory in non-Western educational settings. Third, the study clarifies the mechanisms through which school climate influences teacher innovation. It demonstrates that teacher autonomy and self-efficacy mediate the relationship between school climate and innovation. The interaction between institutional culture and psychological empowerment shapes an educational ecosystem conducive to innovation. Overall, the findings provide a clear analytical framework for understanding how school climate, teacher autonomy, and self-efficacy collectively contribute to teacher innovation, offering valuable insights for educational managers and policymakers seeking to promote innovative practices.

The study also carries important practical implications. First, teacher innovation flourishes in open, supportive, and encouraging school climates. Schools should foster a culture of trust, respect, and collaboration. Establishing transparent communication channels and shared decision-making mechanisms can facilitate professional dialog and reflective practice, creating a psychologically safe environment for innovation. Second, school leaders should implement empowerment mechanisms to provide teachers with substantive autonomy in their work. When teachers perceive institutional trust and empowerment, their sense of responsibility and motivation to achieve instructional goals increases. Finally, targeted professional development and evaluation strategies should be implemented to support teachers’ growth. Professional development initiatives should focus on enhancing teachers’ knowledge and instructional skills, strengthening confidence in their teaching capabilities, and cultivating strong beliefs in their instructional efficacy. Furthermore, school administrators should integrate autonomy and self-efficacy into teacher development frameworks. Only when teachers possess both freedom of action and psychological confidence can sustainable innovation occur.

## 6 Limitations and prospects

This study has certain limitations. First, it used cross-sectional data. Although cross-sectional data can efficiently provide information on school climate, teacher autonomy, self-efficacy, and teacher innovation to explore the relationships among these variables, it is difficult to reveal causal relationships or changes in the impact of school climate on teacher innovation over time. Second, the data in this study were collected from secondary school teachers in Shanghai, China, which is relatively homogeneous and lacks representativeness, limiting the generalizability of the findings. Third, the theoretical model in this study may not have fully captured all the mechanisms through which school climate influences teacher innovation. Finally, this study employed a quantitative research method and investigated the influence of school climate on teacher innovation from the teachers’ perspective. The research methods are relatively single-dimensional, resulting in insufficient depth in exploring the mechanisms affecting teacher innovation.

In future research, longitudinal studies or experimental designs can be conducted based on this study to analyze the issue more deeply. Second, the sample scope should be expanded to include data from multiple countries and regions to carry out comparative studies and enhance the universality of the findings. Third, introduce additional mediating or moderating variables to further refine the theoretical explanatory framework and enhance the model’s explanatory power and robustness. Finally, subsequent studies could adopt mixed-method approaches by combining quantitative data with interviews of teachers and related groups to more comprehensively and deeply reveal the mechanisms through which school climate affects teacher innovation.

## 7 Conclusion

Based on data analysis and research findings, the study draws the following conclusions:

1. The level of teacher innovation in Shanghai, China, is relatively high, and there are significant differences across teaching experience.
2. School climate can significantly and positively predict teacher innovation.
3. Teacher autonomy and self-efficacy play partial mediating roles between school climate and teacher innovation.
4. The chain mediation effect of teacher autonomy and self-efficacy is significant; a positive school climate enhances teacher autonomy and self-efficacy, which in turn improves teacher innovation levels.

## Data Availability

Publicly available datasets were analyzed in this study. This data can be found here: https://www.oecd.org/en/data/datasets/talis-2018-database.html.
